# Hepatocellular Carcinoma to the Right Ventricle

**DOI:** 10.1155/2014/192737

**Published:** 2014-11-30

**Authors:** George R. Marzouka, Apurva Badheka, Alexis P. Rodriguez, Sandra V. Chaparro

**Affiliations:** ^1^Division of Cardiology, University of Miami Miller School of Medicine, 1120 NW 14th Street, Clinical Research Building, Room 1107, Miami, FL 33136, USA; ^2^Department of Internal Medicine, University of Miami Miller School of Medicine, 1611 NW 12th Avenue, Central Building, Room 600D, Miami, FL 33136, USA

## Abstract

Hepatocellular carcinoma (HCC) is the sixth most prevalent cancer in the world, but metastatic disease to the heart is rare. We present a case of a 63-year-old man with history of hepatitis C and cirrhosis, which had progressed to HCC. The patient had undergone two prior liver transplantations. He presented to the hospital complaining of worsening lower extremity edema. His exam was also pertinent for jugular venous distension, a 3/6 crescendo-decrescendo murmur, and hepatosplenomegaly. A transthoracic echocardiogram showed a large irregular lobulated mass in the apex of the right ventricle with a mobile pedunculated component. An MRI of the heart revealed a 4.4 × 3.4 × 4.0 cm mass within the right ventricular apex, which was subsequently biopsied and found to be moderately differentiated HCC with myocardial fragments. The patient opted out of any further therapy, or intervention, and was enrolled in hospice care.

## 1. Introduction

Hepatocellular carcinoma (HCC) is the sixth most prevalent cancer worldwide [1]. However, metastasis of HCC to the heart is rare with prevalence on autopsy of less than 6% [2]. To date, isolated right ventricle metastasis has been reported in only 9 cases [2–4].

## 2. Case Report

**Figure 1:**
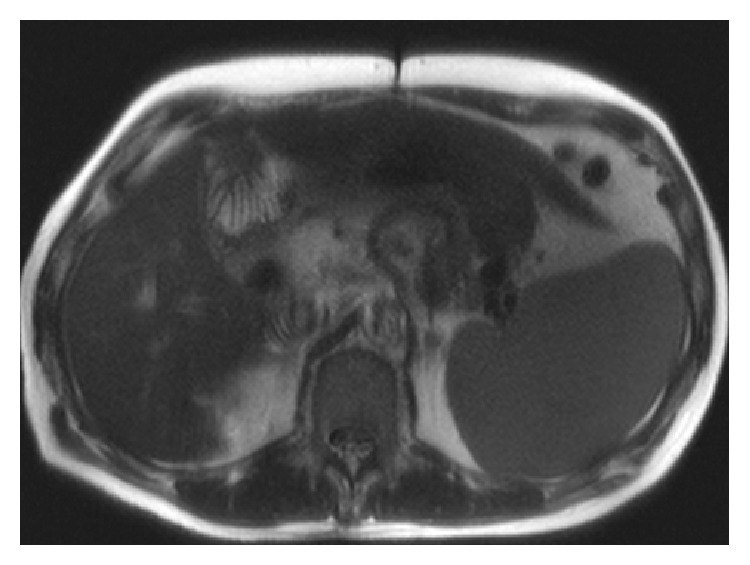
Magnetic resonance of abdomen. Axial view. Limited view with no major evidence of biliary ductal dilatation or filling defect in the visualized portions of the duct. The left lobe of the liver is enlarged. There is also splenomegaly.

**Figure 2:**
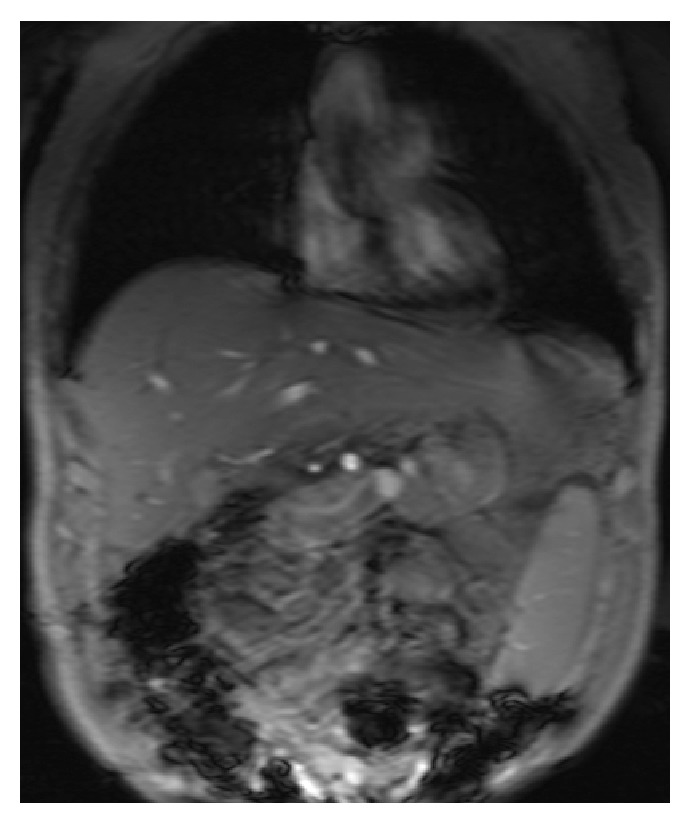
Magnetic resonance of abdomen. Coronal view.

A 63-year-old gentleman presented to our hospital with complaints of lower extremity edema. He had a medical history of thalassemia minor with chronic anemia, hepatitis C, and cirrhosis complicated by moderately differentiated hepatocellular carcinoma (HCC) in 2005 (Figures 1 and 2, MRI of the abdomen). The patient had undergone liver transplantation, which was complicated requiring retransplantation in 2006 secondary to rejection. Over the two years prior to this last admission, he had struggled with chronic ascites and lower extremity edema for which he had been prescribed furosemide. On presentation, he complained of worsening edema to the point of restricting his ambulation due to pain. On physical exam, he had jugular venous distention to the mid neck along with 2+ pitting edema. On auscultation, a 3/6 crescendo-decrescendo murmur was best heard over the left second intercostal space with radiation to the bilateral carotids. His abdomen was benign except for palpable hepatosplenomegaly.

**Figure 3:**
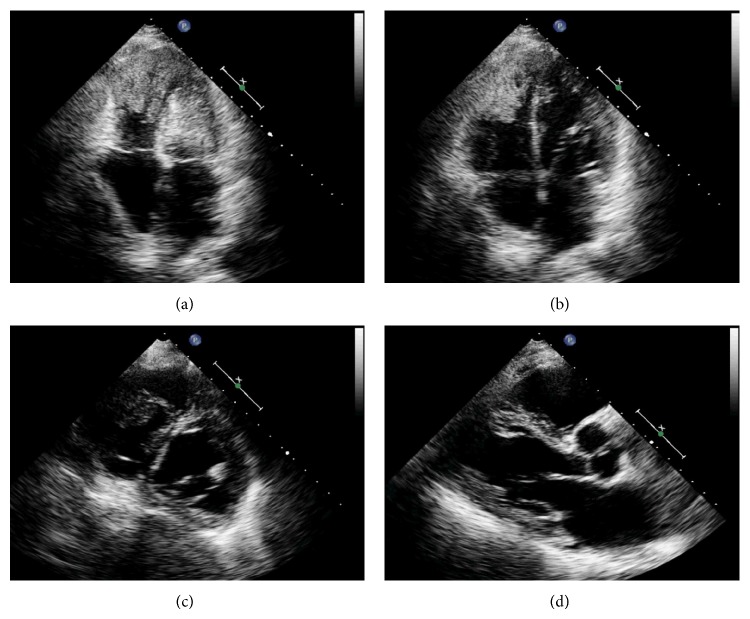
Transthoracic echocardiogram showing irregular mass in the right ventricle.

**Figure 4:**
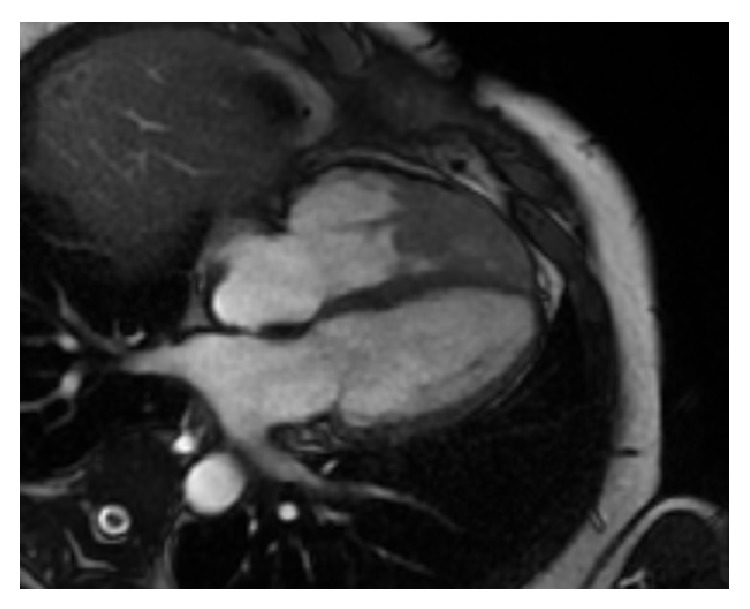
Cardiac MRI showing RV mass. On MRI, the mass was found to involve the apex and mid right ventricle. Ejection fraction on the left ventricle was estimated to 60%. There was also noted turbulence over the aortic valve, suggestive of a possible mass.

**Figure 5:**
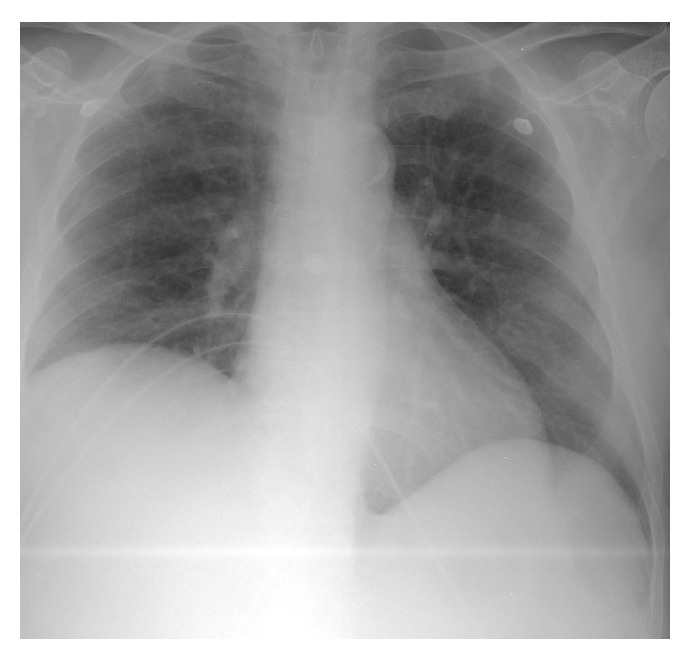
Portable chest X-ray. Showing elevation of right dome of the diaphragm. There is atherosclerotic calcification of aortic arch. The cardiac silhouette is within normal limits.

**Figure 6:**
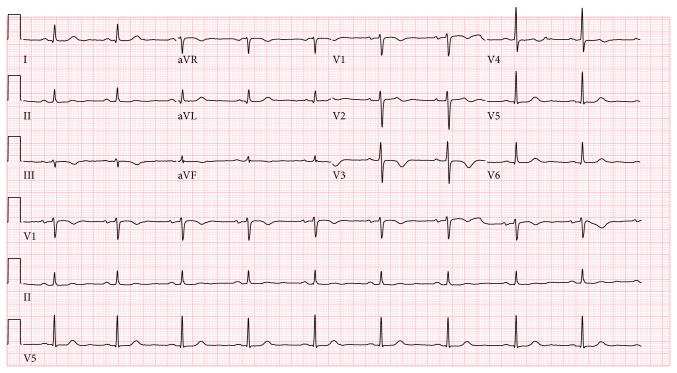
Electrocardiogram. Showing nonspecific T wave abnormalities.

A transthoracic echocardiogram revealed a grossly normal left ventricle with a left ventricular ejection fraction of 60–65%, along with a large irregular lobulated mass occupying the apex of the right ventricle with a round mobile pedunculated component (Figure 3, 2D echo). The right ventricular systolic pressure was elevated at 40–50 mmHg. Moreover, the aortic valve revealed a small round pedunculated mass (3 mm in diameter), which appeared to be attached to the left coronary cusp. A magnetic resonance imaging (MRI) of the heart was performed showing an isointense filling defect within the right ventricular apex on T1 weighed images. T2 weighed images revealed the mass to have a higher intensity signal compared to adjacent myocardium. On imaging, the mass dimensions were estimated at 4.4 × 3.4 × 4.0 cm and involved the papillary muscles (Figure 4). Furthermore, no definitive mass was seen on the aortic valve; however, turbulence of flow suggested underlying valve abnormality. Figures 5 and 6 show the patient's chest X-ray and electrocardiogram, respectively.

**Figure 7:**
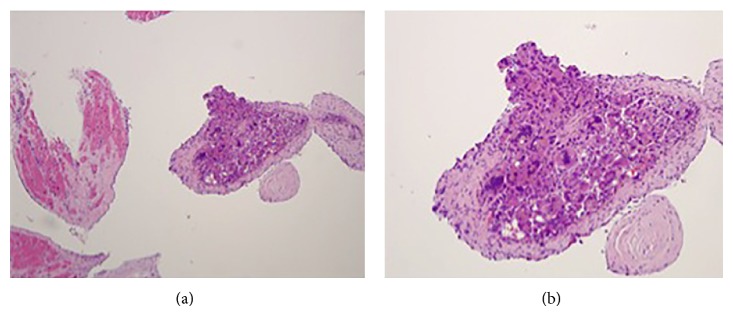
Trichrome stain showing moderately differentiated hepatocellular carcinoma with fragments of myocardium and mild subendocardial fibrosis.

He underwent heart catheterization where four right ventricular biopsy samples were sent to pathology for analysis. On trichrome stain, the mass was determined to be differentiated HCC with fragments of myocardium with mild subendocardial fibrosis (Figure 7). The patient was discharged in hemodynamically stable condition and scheduled to follow up with the transplant clinic to begin therapy for metastatic HCC. However, given the advanced staging of his HCC, liver failure, and functional decline, the patient opted out of further palliative therapy and was transferred to hospice care.

## 3. Discussion

Metastatic tumors are more common than primary neoplasms of the heart and generally involve the myocardium as opposed to the valves or endocardium [2]. They may occur secondary to contiguous extension, lymphatic spread, or hematogenous spread to the myocardium [2]. The most common metastatic cardiac malignancies include bronchogenic and breast carcinomas, lymphomas, leukemias, and various sarcomas [5].

Despite being the sixth most prevalent cancer worldwide, HCC seldom metastasizes to the heart. Such events are rare with a prevalence of less than 6% in one case series of autopsied patients with known HCC [1, 2]. It is estimated that 5–10% of patients with HCC will develop some form of cardiac metastasis [2]. Nevertheless, of those cases, few will represent isolated metastasis of HCC to the right ventricle with involvement of other structures [2]. In the eight other cases reported in the literature, mean survival was approximately 3.67 months reflecting very poor prognosis [2, 3].

In our case, the worsening lower extremity edema could have been confounded with the patient's chronic history of cirrhosis acutely exacerbated with similar complaints. However, the use of echocardiography and magnetic resonance proved to be pivotal in assessing the clinical significance of the auscultated murmur. Furthermore, these imaging modalities helped in determining the location and extent of the intracardiac metastasis. On the other hand, cardiac catheterization provided tissue biopsy for analysis to ultimately reach a conclusive diagnosis.

To the best of our knowledge, there are no clear guidelines for the treatment of cardiac metastatic disease. Since patient prognosis is poor and the cardiac involvement makes the surgical management all the more challenging, surgical resection is generally reserved as a palliative option. Most reports describe prolongation of life for one to fifteen months after successful surgical resection of metastatic HCC in the right heart [2]. Even further, there has been only one reported case of successful transcoronary chemoembolization of a small metastatic HCC mass [6] and one case report of open-heart surgery for a larger mass [3].

In the end, given the complex and challenging nature of the disease and the organ systems involved, the management of these patients should depend on the extent of intra- and extracardiac metastases. The compromised anatomic structures and their physiological roles call for a highly individualized treatment approach. As such, a multidisciplinary team seems the most appropriate option with emphasis on the psychological and palliative support. Moreover, with the continuous changes and improvement in the field of oncology, newer and more promising treatment modalities may emerge. These may indeed prove valuable, if not for curative, at least for palliative purposes.
